# Current and historic HIV-1 molecular epidemiology in paediatric and adult population from Kinshasa in the Democratic Republic of Congo

**DOI:** 10.1038/s41598-020-74558-z

**Published:** 2020-10-28

**Authors:** Marina Rubio-Garrido, José María González-Alba, Gabriel Reina, Adolphe Ndarabu, David Barquín, Silvia Carlos, Juan Carlos Galán, África Holguín

**Affiliations:** 1 HIV-1 Molecular Epidemiology Laboratory, Microbiology and Parasitology Department, Hospital Ramón y Cajal-IRYCIS and CIBEREsp-RITIP, 28034 Madrid, Spain; 2 Virology Section, Microbiology and Parasitology Department, Hospital Ramón y Cajal-IRYCIS and CIBEREsp, 28034 Madrid, Spain; 3 Microbiology Department, Clínica Universidad de Navarra, Navarra Institute for Health Research (IdiSNA), Institute of Tropical Health, Universidad de Navarra (ISTUN), 31008 Pamplona, Spain; 4 Monkole Hospital, Kinshasa, Democratic Republic of the Congo; 5 Department of Preventive Medicine and Public Health, Navarra Institute for Health Research (IdiSNA), Institute of Tropical Health, Universidad de Navarra (ISTUN), Pamplona, 31008 Spain

**Keywords:** Virology, DNA recombination, Molecular evolution, Phylogeny, Evolution, Microbiology, Molecular biology

## Abstract

HIV-1 diversity may impact monitoring and vaccine development. We describe the most recent data of HIV-1 variants and their temporal trends in the Democratic Republic of Congo (DRC) from 1976 to 2018 and in Kinshasa from 1983–2018. HIV-1 *pol* sequencing from dried blood collected in Kinshasa during 2016–2018 was done in 340 HIV-infected children/adolescents/adults to identify HIV-1 variants by phylogenetic reconstructions. Recombination events and transmission clusters were also analyzed. Variant distribution and genetic diversity were compared to historical available *pol* sequences from the DRC in Los Alamos Database (LANL). We characterized 165 HIV-1 *pol* variants circulating in Kinshasa (2016–2018) and compared them with 2641 LANL sequences from the DRC (1976–2012) and Kinshasa (1983–2008). During 2016–2018 the main subtypes were A (26.7%), G (9.7%) and C (7.3%). Recombinants accounted for a third of infections (12.7%/23.6% Circulant/Unique Recombinant Forms). We identified the first CRF47_BF reported in Africa and four transmission clusters. A significant increase of subtype A and sub-subtype F1 and a significant reduction of sub-subtype A1 and subtype D were observed in Kinshasa during 2016–2018 compared to variants circulating in the city from 1983 to 2008. We provide unique and updated information related to HIV-1 variants currently circulating in Kinshasa, reporting the temporal trends of subtypes/CRF/URF during 43 years in the DRC, and providing the most extensive data on children/adolescents.

## Introduction

Several independent zoonotic transmission events from primates to humans around the beginning of the twentieth century^1^ in Central and West Africa gave rise to Acquired Immune Deficiency Syndrome (AIDS) in the human population, spreading quickly worldwide^2,3^. The HIV epidemic is the result of two types of viruses: HIV-1 and HIV-2, which are closely related to SIV_*ptt*_ and SIV_*sm*_ respectively^4,5^. HIV-1 causes most HIV infections worldwide and has been divided into 4 groups (M, N, O and P)^3,6–8^, but the global HIV epidemic is related to group M, which has been subdivided into 10 subtypes (A-D, F–H, J-L)^1,9,10^, at least 100 Circulating Recombinant Forms (CRF)^11^ and uncountable Unique Recombinant Forms (URF). HIV-1 diversity occurred in the early steps in the human adaptation of SIV_*ptt*_^12^.

Since HIV-1 variability can impact transmission, pathogenesis and disease progression^13–15^, HIV monitoring^16,17^, resistance pathways^18^, cognitive impairment^19^, and HIV vaccine development^20^, an accurate HIV-1 variant identification is needed^21^. It can enable a better understanding of global HIV expansion^22,23^ and to infer the evolutionary possibilities in the selection of new variants, such as the recently described subtype L^10^. HIV-1 molecular surveillance studies are globally important and of interest in any setting. However, it is more interesting to analyze HIV molecular diversity in an area with a high viral diversity, especially if the place is the origin of a HIV-1 pandemic, where the virus has been circulating for a longer period and new, more pathogenic or transmissible variants could have arised. HIV-1 pandemic originated in Central Africa^24^, specifically in the DRC, Kinshasa being the epicenter^12^, spreading to neighboring cities or countries^25^.

The most ancestral available HIV-1 sequences were recovered from Kinshasa in 1959 (ZR59, near the ancestral node of subtypes B and D)^26^, 1960 (DRC60, subtype A)^1^ and 1966, (DRC66, subtype C)^27^. Subtype B also emerged in Kinshasa and was introduced into the Caribbean region via Haiti around 1966 by human migration and later disseminated to other regions, such as the United States of America, Europe, Asia, Latin America, and Australia^23^.

Accordingly, Kinshasa is one of the most relevant places to carry out surveillance programs to increase the knowledge of HIV molecular epidemiology and viral evolution. In that context, our study presents the most recent data related to current circulating HIV-1 variants in *pol* in Kinshasa during the 2016–2018 period, identifying transmission clusters and updating the genetic diversity. In addition, we present a complete review of HIV-1 variant trends in the DRC over 43 years (1976–2018) after using all the historical *pol* sequences available in Los Alamos HIV Sequence Database (LANL) from samples collected in the DRC. We reclassified all HIV-1 variants after new phylogenetic analysis in all downloaded *pol* sequences, including the most recent subtype L and new CRF described in the last years. Later we studied the temporal trends of HIV-1 variants in the DRC and Kinshasa considering the sampling year of each sequence.

## Results

### Current update of HIV molecular epidemiology in the DRC

We obtained viral *pol* sequences (115 protease, PR, 139 retrotranscriptase, RT and 115 integrase, IN) from 165 (48.5%) of 340 HIV-infected children/adolescents (55, 33.3%) and adults (110, 66.7%) from two hospitals in Kinshasa (DRC) with dried blood specimens (DBS) collected during 2016–2018 (Table 1). Thus, our study was done in 165 patients with available *pol* sequence. The median age [IQR] at DBS collection for children and adults was 14.4 [11.4–16.8] and 43 [34–53.5] years old, respectively. Around 75% of subjects with available *pol* sequence were under antiretroviral treatment (ART) and 8 out of 10 presented more than 1000 HIV-1 RNA copies/ml at sampling (Table 1).

**Table 1:** Main characteristics of HIV-1 infected population from Kinshasa (DRC) with collected DBS in 2016–2018.

	Children/adolescents (%)	Adults (%)	Total (%)
Subjects with *pol* sequence	55 (33.3)	110 (66.7)	165 (100)
Female	28 (50.9)	64 (58.2)	92 (55.8)
Age at DBS collection (years), median [IQR]	14.4 [11.4–16.8]	43 [34–53.5]	32.4 [16.2–48.5]
**ART exposure**			
ARV naive	0	11 (10)	11 (6.7)
ART	55 (100)	69 (62.7)	124 (75.2)
Unknown	0	30 (27.3)	30 (18.2)
**HIV-1 viraemia>1000 HIV-1-RNA copies at sampling (data known in 55 children and in 103 adults)**			
In 1 DBS dot [range]	19/55 (34.5) [1260–16,700]	51/103 (49.5) [1200–132,000]	70 (42.4) [1200–132,000]
In 1 ml plasma* [range]	49/55 (89.1) [1351–469,320]	86/103 (83.5) [1008–3,901,478]	135 (81.8) [1008–3,901,478]
**Available** ***pol*** **HIV-1 sequences**			
PR	36 (65.5)	79 (71.8)	115 (69.7)
RT	49 (89.1)	90 (81.8)	139 (84.2)
IN	40 (72.7)	75 (68.2)	115 (69.7)
**Combination of sequences by regions**			
Only PR	0	1 (0.9)	1 (0.6)
Only RT	5 (9.1)	8 (7.3)	13 (7.9)
Only IN	6 (10.9)	19 (17.3)	25 (15.1)
PR+RT	10 (18.2)	26 (23.6)	36 (21.8)
RT+IN	8 (14.5)	4 (3.6)	12 (7.3)
PR+RT+IN	26 (47.3)	52 (47.3)	78 (47.3)
**HIV-1 variants at** ***pol***			
Subtype B	1 (1.8)	0	1 (0.6)
Non-B subtypes	28 (50.9)	70 (62.4)	98 (59.4)
CRF	2 (3.6)	19 (17)	21 (12.7)
URF	22 (40)	17 (14.5)	39 (23.7)
U	2 (3.6)	4 (3.6)	6 (3.6)

DRC, Democratic Republic of Congo; DBS, dried blood specimens; ART, antiretroviral treatment; ARV, antiretroviral drugs; PR, protease; RT, retrotranscriptase; IN, integrase; *pol*, HIV-1 *pol* coding region; CRF, circular recombinant forms; URF, unique recombinants forms; U, unknown variant, not ascribed to pure subtype, CRF or URF.

*Estimated HIV-1-RNA copies considering hematocrit^70,71^.

**Figure 1:**
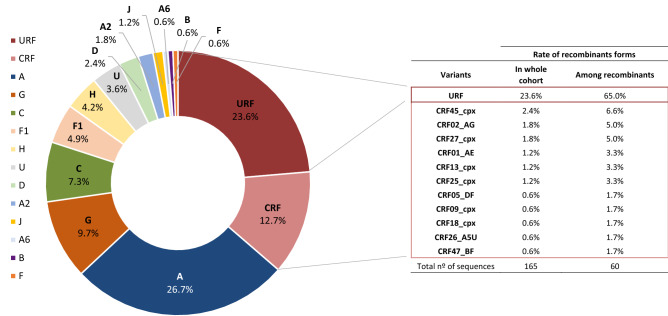
Current molecular epidemiology characterization in our HIV-1 study cohort from Kinshasa (DRC) with collected DBS in 2016–2018. Percentages calculated considering new HIV-1 *pol* sequences recovered from DBS of 165 HIV-1 infected subjects from Kinshasa during 2016–2018.

**Figure 2:**
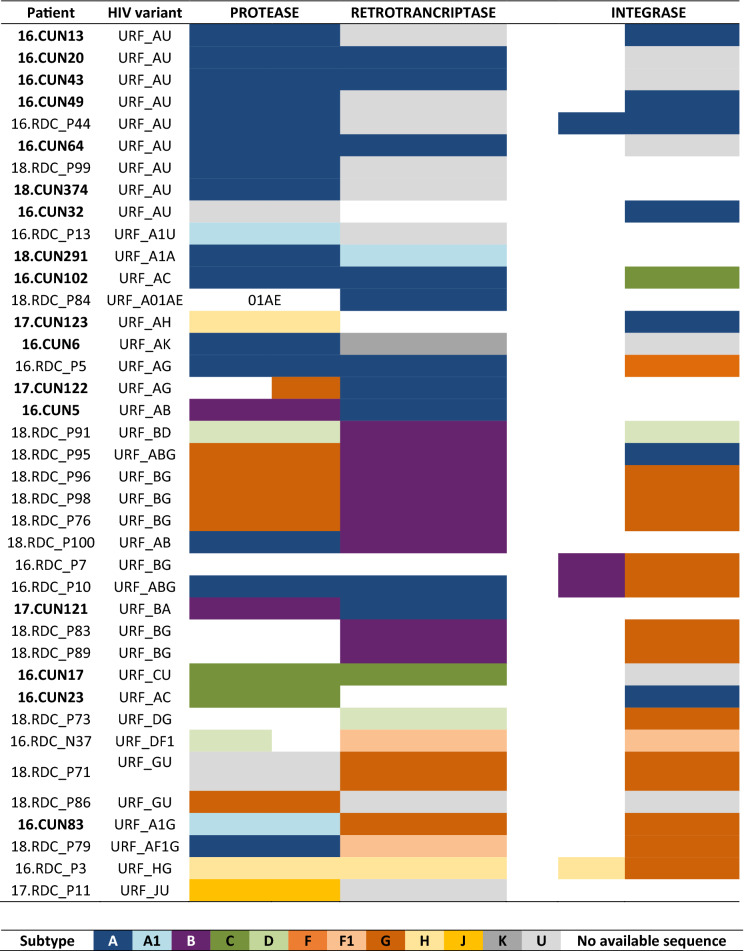
Recombination structure of the available *pol* sequences assigned to URF and recovered from 39 HIV-1 infected subjects in Kinshasa (2016–2018). This figure represents a simplified URF structure. In bold, viruses collected from adults. The remaining, viruses collected from children/adolescents.

From the historical database, we recovered 5672 group M *pol* sequences without gaps or ambiguities available in LANL, 4090 of them being assigned to HIV-1 pure subtypes (300 A, 2476 B, 1133 C, 72 D, 48 F, 45 G, 7 H, 5 J, 1 K, 3 L) and 1582 circulating recombinants forms CRF_01 to CRF_99. As expected, most (99.4%) viral sequences from the 165 subjects were classified as HIV-1 non-B variants in *pol* coding region. Among them, 59.4% were 9 pure non-B subtypes, 36.4% recombinant forms (23.7% URFs and 12.7% CRFs) and 3.6% unique unclassificable (U) sequences. The most frequent HIV-1 variant was subtype A (26.7%), followed by subtypes G (9.7%) and C (7.3%) (Fig. 1 and Sup. Fig. 1). The most prevalent CRF were CRF45_cpx (2.4%), CRF02_AG (1.8%) and CRF27_cpx (1.8%) in our cohort. Interestingly, we identified for the first time the CRF47_BF variant in the DRC, infecting an 8 year-old child. Among recombinant forms, URF accounted for 65% of infections in our study cohort (2016–2018). These URF were in circulation in Kinshasa during 2016–2018. Recombination patterns in the analyzed *pol* fragments assigned to URF among the 39 subjects is described in Fig. 2. When specific HIV-1 variants were compared between age groups, adults presented a greater number of different CRF (11 variants) than children/adolescent group (2 CRF: CRF27_cpx and CRF47_BF). Althought the children/adolescent group presented less diversity, they had almost 3 times as many URF infections than adults (40% vs. 14.5%; *p* < 0.05). The rate of pure subypes was similar across both groups.

### Cluster identification between non-epidemiologically related adults and children/adolescents

Among the 165 *pol* sequences from Kinshasa we identified 4 independent transmission clusters (Table 2 and Sup. Fig. 2–5) supported by 100% bootstrap, presenting recent transmission (genetic distance lower than 0.01) in three of them: cluster 1, cluster 3 and cluster 4. Infected individuals from clusters 1, 2 and 3 carried subtype A *pol* sequences, although they were not ascribed to previously characterized sub-subtypes A or CRF including A at *pol* coding region. They appeared in 3 different branches in PhyML trees after including all LANL sequences ascribed to subtype A and their sub-subtypes circulating in the DRC since 1976 (Sup. Fig. 2–4). Subjects from cluster 4 harbored subtype H viruses, clustering apart from other subtype H LANL sequences from the DRC (Sup. Fig. 5). Table 2 shows the epidemiological data collected from clinical reports of each patient. All clusters included 2 subjects, except cluster 1 including a 34.7 year-old woman, a 6.2 year-old boy and a 14.3 year-old adolescent male with no reported epidemiological link (Sup. Fig. 2). Clusters 2 and 3 included two adults and cluster 4 two children.

**Table 2:** Clusters identification in the studied population from Kinshasa in the 2016–2018 period.

Cluster nº	Patients data				Cluster data				
	Involved subjects	Group of age	Age at sampling	Gender	HIV-1 variant	Analized nts	Bootstrap (%)	Genetic distance**	PhyML tree
1	16.RDC_P33	Adolescent	14.3	M	A*	1270	100	0.02***	Sup.Fig 2
	18.RDC_P64	Child	6.2	M					
	18.CUN288	Adult	34.7	F					
2	16.CUN24	Adult	57.7	F	A*	1279	100	0.05	Sup.Fig 3
	16.CUN82	Adult	62.3	M					
3	16.CUN28	Adult	50.6	F	A*	649	100	0.003	Sup.Fig 4
	16.CUN32	Adult	37.6	F					
4	16.RDC_P3	Child	10.2	M	H	1269	100	0.005	Sup.Fig 5
	17.RDC_P49	Child	9.7	F					

Nts, nucleotides; Sup, supplementary; Fig, figure; M, male; F, female.

*Subtype A sequences not ascribed to previously characterized sub-subtypes A or CRF including A at *pol *coding region recovered from patients in the DRC with available LANL sequences.

**We considered recent transmission when genetic distance was lower than 0.01.

***Genetic distance <0.01 comparing P64 and CUN288.

### Transmission networks involved a common recombinant fragment in recent paediatric/adolescents sequences from Kinshasa

Among the 165 new sequences (2016–2018), we found the presence of a transmission network, including two different clusters (RecG and RecB) of 17 viruses sharing one* pol* fragment (Table 3 and Sup. Fig. 6). Interestingly, all viruses involved were collected from paediatric patients with no epidemiological linkage according to clinical reports. Cluster RecB included viruses sharing a fragment of 599 nt in RT (positions 2935–3534 in HXB2) assigned to subtype B, in 8 patients ranging from 9 to 18 years-old. Cluster RecG included viruses from 9 adolescents from 10 to 19 years-old sharing a fragment in IN (positions 4390–5005 in HXB2) of 615 nt assigned to subtype G (Table 3). Of note, one *pol *sequence from a 19 year-old female (18.RDC_P83) shared fragments of both clusters (Sup. Fig. 6). The common progenitor viruses for clusters RecG and RecB using PhyML trees were not found in all 2802 LANL sequences from the DRC. We also observed a recombination event at protease coding region involving viruses from 2 adults (subtype A, CUN131 and subtype H, CUN127), leading to URF_A virus infecting CUN123 (Sup. Fig. 7).

**Table 3:**
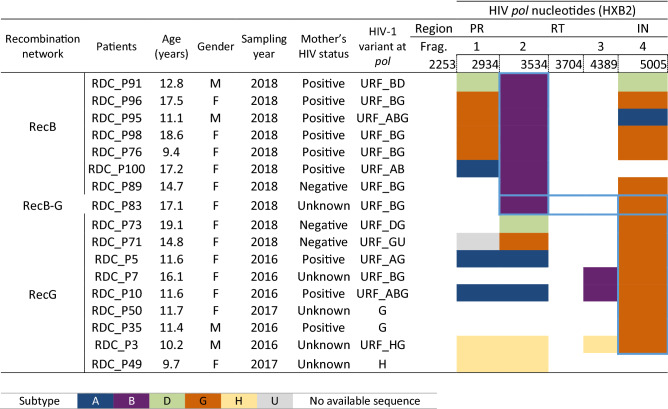
Description of recombination transmission net found among the 165 HIV-1 variants collected in Kinshasa from 2016 to 2018.

RecB, recombination network sharing 599 nts subtype B fragment in RT; RecG, recombination network sharing 615 nts subtype G fragment in IN; M, male; F, female; frag., fragment. Analysed *pol* fragment (numbering according to HXB2): 1, positions 2253-2934 (in protease); 2, positions 2935-3534 (in retrotranscriptase); 3, positions 3704-4389 (in RNase H); 4, positions 4390-5005 (in integrase).

### Reclassification of HIV-1 variants with LANL sequence circulating in the DRC since 1976

**Figure 3:**
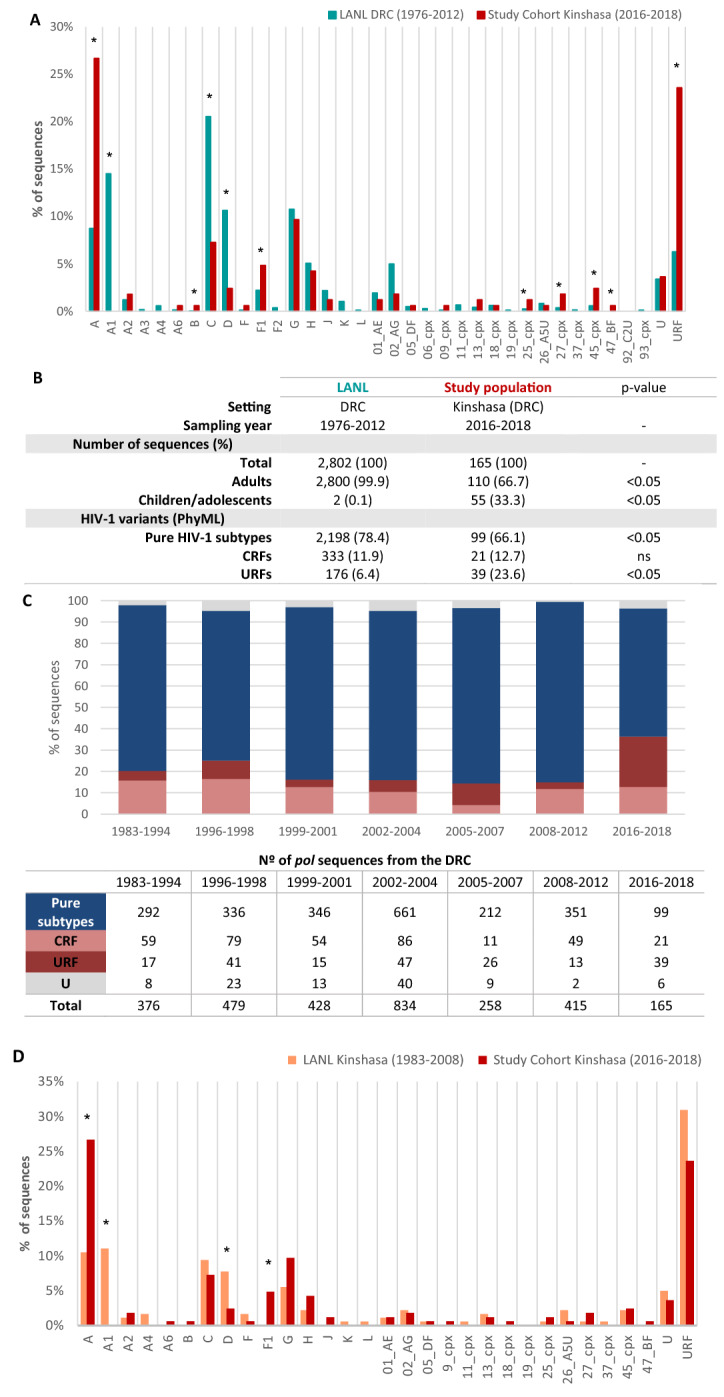
Comparison of HIV-1 variants in the study cohort (2016–2018) versus LANL sequences from the DRC (1976–2012) and Kinshasa (1983–2008). (**A**) Comparison of HIV-1 variants in the study cohort from Kinshasa (2016–2018) versus DRC sequences available in LANL (1976–2012). (**B**) Comparison of data between study cohort and LANL. (**C**) Evolution of pure subtypes and recombinants forms in the DRC by periods of time during 1983–2018. Panel C excludes *pol* sequences from the DRC sampled during 1977–1982, 1986, 1995, 2009, 2010, 2013–2015, due to their absence in LANL database. We clustered all sequences sampled during 1983–1994 as an “early period” to avoid biases due to the non-representative number of *pol* sequences each year within that period. Panels B and C exclude 214 LANL sequences from the DRC with unknown sampling time collected before 2012. (**D**) Comparison of HIV-1 variants distribution circulating in Kinshasa during 1983–2008 versus 2016–2018. For analysis we used the 181 *pol* sequences with available GenBank accession number from samples collected in that city during 1983–2008 versus the new 165 *pol* sequences collected during 2016–2018 reported in this study. Cpx, complex; CRF, circulating recombinant form; URF, unique recombinant form. *p value < 0.05.

In order to increase the number of *pol* sequences from the DRC, we downloaded all 2802 available *pol* sequences in LANL, which had been collected since 1976 to 2012, mainly (99.9%) from adults (Fig. 3). Then, since new HIV-1 variants have appeared in the recent years, we reclassified the 2641 pure subtypes and CRF, excluding 161 URF, performing the same PhyML analysis and reference sequences set in the historic LANL database from the DRC as in our sequences collected in Kinshasa from 2016–2018. Interestingly, we found some discrepancies in the HIV-1 variant classification in 8.8% (198 sub-subtype A1 and 34 U) of them, which had probably been misclassified due to less available reference HIV-1 sequences and to the absence of new subtypes and new CRF when they were classified. The new PhyML analysis revealed that 198 (32.9%) of the 602 sequences originaly appointed as sub-subtype A1 in LANL were, in fact, other HIV-1 variants, 101 being subtype A (51%), 88 CRF02_AG (44.4%), 3 CRF11_cpx (1.5%), 3 CRF25_cpx (1.5%), 1 sub-subtype A6 (0.5%), 1 sub-subtype F1 (0.5%) and 1 U (0.5%). Among the 34 (26.6%) of 128 U LANL sequences from the DRC, 12 turned out to be subtype A (35.3%), 4 sub-subtype F1 (11.8%), 3 sub-subtype A4 (8.8%), 3 sub-subtype A5 (8.8%), 3 subtype D (8.8%), 3 CRF18_cpx (8.8%), 2 subtype K (5.9%), 1 CRF02_AG (2.9%), 1 subtype G (2.9%), 1 subtype H (2.9%), and 1 (0.5%) subtype L. Thus, one third (32.9%) of sub-subtype A1 and a quarter (26.6%) of U LANL sequences from the DRC had been previously misclassified.

### Changes in the HIV-1 molecular epidemiology in the DRC (1976–2018) and in Kinshasa (1983–2018)

In order to analyze the HIV-1 molecular epidemiology evolution over time in the DRC, we compared the current HIV-1 variants in *pol* circulating in Kinshasa (2016–2018) with the 2588 *pol* sequences previously reported in the country with known sampling year (1976–2012) and newly reclassified in this study. Globally, a significant reduction (*p* < 0.05) in the percentage of pure subtypes and simultaneously an increase of URFs (*p* < 0.05), were observed after comparing both periods, whereas the proportion of CRFs remained similar across both sequence sets (Fig. 3A, B). The evolution of pure subtypes and recombinant forms in the DRC across a three-year period until 2018 is shown in Fig. 3C.

HIV-1 variant distribution varies greatly between provinces and it is not possible to compare the diversity in Kinshasa with that of the whole country. We therefore performed a new analysis using all available *pol* sequences deposited in LANL recovered from samples collected in Kinshasa after identifying the accession numbers in the corresponding papers, recovering 181 *pol* sequences from different subjects. We then compared the HIV-1 variant distribution in Kinshasa from the 181 samples collected exclusively in the city during 1983–2008 (94.5% of them sampled during 2007–2008) versus the new 165 *pol* sequences collected during 2016–2018 in Kinshasa and newly reported in this study. We observed during 2016–2018 a significant increase in subtype A (10.5% vs. 26.7%, *p* < 0.05) and sub-subtype F1 (0% vs. 4.8%, *p* < 0.05) and a significant reduction in sub-subtype A1 (11% vs. 0%, *p* < 0.05) and subtype D (7.7% vs. 2.4%, *p* < 0.05) compared to variants from Kinshasa circulating from 1983 to 2008, whereas the proportion of CRFs remained similar across both sequence sets (12.1 vs. 13.2%, respectively). We detected CRF47_BF virus for the first time in the country using *po*l sequences collected after 2016. URF tended to decrease over time (31% vs. 23.6%). The remaining variants remained stable over time (Fig. 3D).

### Differences on genetic diversity across *pol* sequences sets from the DRC

**Figure 4:**
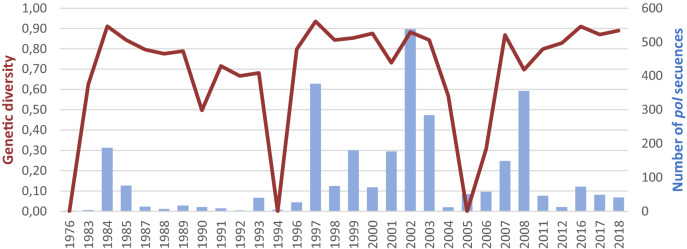
Temporal trends of genetic diversity across years in HIV-1 *pol* sequences from the DRC. Bars in blue, available *pol* sequences from the DRC with known sampling year. LANL database did not include *pol* sequences from the DRC sampled during 1977–1982, 1986, 1995, 2009, 2010, and 2013–2015.

The global genetic diversity across the 2802 LANL *pol* sequences (1976–2012) versus our 165 *pol* sequence set under study (2016–2018) reported similar values (0.90 vs. 0.91) (Table 4). However, considering specific HIV-1 variants, LANL set presented higher genetic diversity in pure-subtypes (0.84 vs. 0.76), whereas diversity of CRFs was higher since 2016 (0.78 vs. 0.89). When the genetic diversity was compared into the most recent sequences, according to age group, it was higher in children/adolescents than adults (0.92 vs. 0.88), mainly in URF variants (0.93 vs. 0.80), whereas in CRFs the diversity was higher in adults versus children/adolescents (0.88 vs. 0.50) (Table 4). Figure 4, shows the high genetic diversity over time in *pol* sequences sampled in the DRC since the 1976 (year of first available *pol* sequence) to 2018, with some variations by periods of time.

**Table 4:** Genetic diversity by study groups in HIV-1 *pol* sequences from the DRC.

	LANL DRC 1976–2012	Study cohort Kinshasa 2016-2018		
		Total	Children/adolescents	Adults
Total	0.90 (n = 2802)	0.91 (n = 165)	0.92 (n = 55)	0.88 (n = 110)
Pure subtypes	0.84 (n = 2198)	0.76 (n = 99)	0.73 (n = 29)	0.75 (n = 70)
CRF	0.78 (n = 333)	0.89 (n = 21)	0.50 (n = 2)	0.88 (n = 19)
URF	0.95 (n = 176)	0.92 (n = 39)	0.93 (n = 22)	0.80 (n = 17)

CRF, circulating recombinant form; URF, unique recombinant form; n, number of *pol *sequences; LANL, Los Alamos HIV Sequence database.

## Discussion

The DRC is known as the origin of HIV-1 pandemic and the epicenter for the selection and spreading of many HIV-1 variants to neighboring countries^28^. As a consequence of high HIV-1 genetic heterogenicity (probably the highest diversity rate in the world), the accurate phylogenetic reconstructions have not been easy to interpret^29^. For this reason, our first approach was to reanalyze the available HIV-1 sequences deposited in LAN from the DRC, observing 8.8% of misclassified sequences according to the new reclassification of HIV-1 variants and phylogenetic programs used in this work.

Global geographical patterns in HIV-1 variants distribution are changing over time due to several factors, such as population movements or the dense transmission networks. These factors are contributing to an unpredictable HIV-1 pandemic^30,31^. Consequently, a continuous and accurate molecular epidemiology surveillance is necessary for increasing our knowledge about the evolving HIV-1 epidemic, especially in those geographical areas with high genetic diversity rate, where multiple HIV-1 variants co-circulate^20,32^, such as the DRC. In these settings, the selection of new recombinant forms is easier and the viral evolution could be faster than in those regions with low diversity of HIV-1^33^.

Due to the lack of updated data on HIV molecular epidemiology in the DRC, we present the information of 165 *pol* sequences obtained in adult and paediatric populations, representing the most recent data on circulating HIV-1 subtypes and recombinant forms in Kinshasa ranging 2016–2018. We also compared the temporal trends of HIV-1 variants in the DRC for a 43-years period, based on 28^1,10,24,28,29,34–56^ published studies. Sup. Table 1 shows the sampling year, sample type, study population, number of analyzed sequences, coding regions, subtyping method and sequence submission to databases in each 28 published studies characterizing HIV-1 variants in the DRC. Since HIV is prone to recombination during retrotranscription, high levels of recombinant forms after coinfections or superinfections are expected, especially in places with high viral diversity, such as in the DRC^24,34^. In this scenario HIV-1 can accelerate adaptation to the host, favouring emerging variants with unknown pathogenicity^57^.

Previous studies have also reported heterogeneous geographic distribution of group M variants across regions in the DRC^58^, with high prevalence of recombinants. However, HIV recombinant rates in our study and previously reported in the country could be underestimated since most of them were classified by using short partial genome sequences (Sup. Table 1) and not full genomes. Thus, the recombinant rate in the DRC could be even higher if more viral regions are assessed. Although the biological and clinical impact remains to be clarified, there is growing evidence that recombination has played a significant role in the early history of the HIV-1 pandemic and in viral evolution^59^. We reported the first identification of CRF47_BF in Africa and the DRC, a recombinant variant first described in Spain^60^. It would also suggest a possible importation of subtype B to the DRC.

This study confirmed subtype A as the most prevalent subtype, which has maintained a high rate of infections in the DRC over the last three decades^61^, explaining the high rate of URF carrying subtype A fragments circulating in Kinshasa described in the last years. Kinshasa has been proposed as the origin of A, G and F1 subtypes^28^, common found HIV-1 variants in our series. Although subtype D was associated with faster disease progression^13^, was also initially emerged in Kinshasa, its prevalence decreased in the city during 2016–2018 compared to previous years. Although a significant increase of subtype C was reported from 1997 to 2002 in Kinshasa^38^, in other countries close to the DRC^62^ and worldwide^15^, we observed a similar prevalence of this variant in Kinshasa comparing the two time periods under study. The reasons for these findings are unknown.

We also found that almost 4 out of 10 variants circulating in Kinshasa during both periods (1983–2008 and 2016–2018) carried recombinant sequences in *pol*. When CRFs and URFs diversities were analysed according to age of patients, CRFs diversity in *pol* was higher in adults and URF diversity higher in children/adolescents in the last period. The trends of HIV-1 genetic diversity should be further explored in the coming years, also including other HIV-1 genes. The high misclassification in sub-subtype A and U *pol* sequences from the DRC deposited in LANL reveals the need for reclassification by phylogeny with updated reference sequence sets and new reported HIV-1 variants.

Phylogenetic approaches and distance-based analysis represent the most common strategy to identify recent transmission networks^63^, and it could be useful to identify other HIV infected and uninfected persons at highest risk of transmission who could benefit from HIV prevention interventions^64^. Some reports have demonstrated that genetic distance restrictive thresholds between 0.01 and 0.02 substitutions per site have been more strongly associated with probable transmission partners than traditional epidemiological connections^65–67^, and a distance of 0.015 could serve as a use proxy for epidemiological relatedness in a surveillance setting^66^. Our study identified that 3 out of 4 transmission clusters were recent (genetic distance thresholds 0.01–0.02^65–67^). We implemented a strategy based on traceability of genetic fragments, to know the potential network among infected people (Sup. Fig. 6). This strategy, “recombination network”, gave us a dense net and probably a more complete view. The recombination network found involving *pol* sequences from 17 children/adolescents with no epidemiological link according to clinical data confirmed the important role of recombination in an HIV pandemic and the importance of common ancestor identification to understand recombination origin and spread. As the children are overrepresented in this network, we should suspect that this network is even denser.

This study has some limitations that need to be considered. Firstly, samples from 2016–2018 were collected in two reference hospitals in Kinshasa, which cannot be fully representative of the situation in a city and even more at a country-wide level^58^. However, the inclusion of all *pol* sequences from LANL collected in the country over more than 4 decades, from the general population and risk groups, provides a good overview of the huge diversity of the country. A second limitation is the length of recovered *pol* sequences, which differed across samples, complicating bioinformatic analysis. A third limitation is that some reports in the DRC did not submit sequences to databases, and they were not available in LANL (Sup. Table 1). These reported HIV-1 variants could therefore not be reevaluated by new PhyML analysis to be included in the temporal tend analysis of HIV-1 variants. Thus, we encourage the necessity of sequence submission in all HIV epidemiology studies worldwide before publication, and the inclusion of the sampling year in all submitted sequences, absent in 214 (8%) of *pol* sequences downloaded from LANL in that country. The lack of routine resistance testing during clinical follow-up of HIV-infected subjects in the DRC limits the *pol* sequences availability in the DRC. Furthermore, the lack of complete epidemiological information from subjects involved in transmission or recombination clusters limits full understanding of a cluster´s origin. Finally, 51.5% of DBS did not yield any viral sequences, possibly due to the low viral load (40–1000 HIV-1 RNA copies/dot) in more than half of specimens and to the low plasma volume in the 2 dots used for HIV-1 RNA extraction, limiting the ability to get positive PCR amplifications of HIV in these samples.

Since none of sequences downloaded form LANL was sampled after 2012, our sequence set was the most recent in the DRC to date and the highest in number of analyzed *pol* sequences in Kinshasa. Our study reinforces the use of dried blood as a field-friendly, useful, convenient and alternative specimen type to whole blood or plasma in HIV molecular epidemiology surveillance studies in developing countries or settings with limitations as regards the collection, storing, transportation of plasma or when low blood volume is available^68^. Only 3 of 27 studies reporting variants in the DRC^48,50,51^ had used DBS for HIV-1 variant characterization (Sup. Table 1). Although two previous studies reported the full HIV-1 genome (including *pol*) from a vertically infected 12-month-old baby^54^ or *gag* sequences from 15 children in Kimpese, rural DRC^53^, our study presents the most extensive data regarding HIV-infected children and adolescents in the country. The updated high genetic diversity observed in the DRC also represents a real challenge for future vaccine development and for efficiency of antiretroviral treatment, diagnostic and monitoring tests of HIV infection^69^.

In conclusion, we report the most recent data related to HIV-1 variants circulating in Kinshasa, the geographical origin of the pandemic, and unique and updated information on the temporal trends of HIV-1 subtypes, CRF and URF in the DRC during 43-year period (from 1976 to 2018) and in Kinshasa from 1983–2018 after reclassification of several available LANL sequences using phylogenetic approach. The data provided increase and update the knowledge of HIV molecular epidemiology in the DRC. Active transmission clusters were detected, and a new strategy offers us a more complete view of transmission networks. Of concern is an overrepresentation of children was observed in the recombination network. Continued molecular surveillance will be essential to determine and trace rare unique recombinant forms or emerging strains of HIV in the country.

## Material and methods

### Sample collection and viral load quantification

From 2016 to 2018, 2 DBS cards were collected from 340 patients under clinical follow up at Monkole and Kalembelembe Hospitals (Kinshasa, DRC), 71 HIV-infected children (0–14 years) and adolescents (15–21 years) and 269 HIV-infected adults (more than 22 years). DBS preparation and viral load quantification was performed using Cobas Ampliprep/Cobas Taqman HIV-1 test v2.0 (Roche) as previously reported^50^ considering hematocrit^70,71^.

### Amplification and sequencing

For HIV-1 variant characterization, viral RNA was extracted from 2 DBS dots using NucliSENS EeasyMAG automated platform (BioMerieux) or manual High-Pure Viral Nucleic Acid kit (Roche). The encoding regions PR, RT, and/or IN at HIV-1 *pol* gene were amplified by RT-PCR and nested-PCR using primers designed by WHO^72^ and ANRS^73^. PCR amplicons were purified using the Illustra ExoProStar 1-Step (GE Healthcare Life Sciences, Little Chalfont, UK) and sequenced by Macrogen Inc. (Geumchun-gu, Seoul, Korea). Viral sequences included the complete HIV-1 PR (codons 1–99), partial RT (codons 1–335/440) and IN (codons 1–285).

### Accession numbers

PR, RT and IN HIV-1 sequences were submitted to GenBank (www.ncbi.nlm.nih.gov/genbank) with the following accession numbers: MH920378-MH920435, MN530990-MN531082, MN998509-MN998523.

### Phylogenetic analysis

Nucleotide sequences were translated and aligned using the ClustalW algorithm implemented in MEGA6. For HIV-1 subtype characterization we used sequences available in Los Alamos HIV Sequence Database (LANL: https://www.hiv.lanl.gov), annotated by country of origin and date. Reference sequences from all HIV-1 variants described to date with available sequence were included (groups, subtypes, sub-subtypes, CRFs). One HIV-1 group N sequence was used as an outgroup. Sequences from groups P/O/N were also downloaded to discard non group-M infections. To alleviate the burden of computer-time required to reconstruct large phylogenies fast algorithms, phylogenetic trees (PhyMLtree) were reconstructed by maximum-likelihood (ML) method with RAxML v8.0 (Randomized Axelerated Maximum Likelihood)^74^ using the general time reversible plus proportion of invariable sites plus gamma distribution parameter (GTR + I + G) evolutionary model. To estimate the bootstrap values on the inferred topology by RAxML, Shimodaira-Hasegawa (SH) test using FastTree program was used (support > 90%)^75^. Sequences not clustering with any variant were analyzed using Recombination Detection Program (RDP3v4.13)^76^, identifying the subtypes involved in eventual recombination events and hypothetical recombination breakpoints. To further confirm the detected putative recombination events, new phylogenetic analyses were performed using the sequence fragments assigned to different subtypes according to the proposed breakpoint position(s) defined by RPD3. The topologies obtained with each fragment were compared to SH, expected likelihood weight, and Kishino-Hasegawa tests using the TREE-PUZZLE 5.2 program. In the positive cases, the recombinant sequences were redefined as URFs; otherwise, sequences with a most recent common ancestor to subtypes sub-subtypes A1-A6 or F1-F2 were identified as subtypes A or F, respectively. The remaining cases were appointed as U. Since it was not possible to sequence the same fragment for all sequences, different regions were analyzed separately.

Transmission clusters in our 165 study sequences from Kinshasa (2006–2018) were defined as viral sequences belonging to the same subtype/CRF/URF and grouped into a single and well-supported cluster (monophyletic clade) with 100% bootstrap values. When only partial *pol* sequences could be recovered from new samples, each available sequence fragment present in all viruses from the cluster was used for analysis separately. In this way, new recombination events were detected. Sequences with different recombination events but sharing some recombinant fragment and common progenitor virus were considered as transmission networks.

Genetic diversity (*D* = 1 − ∑*f*^2^) of HIV variants in the DRC was analyzed over time, a measure of variability that takes into account the frequencies (*f*) of all variants. For that purpose, all 2802 *pol* LANL sequences from the DRC were downloaded and reclassified as previously described. Among them, 2588 (92.3%) reported the sampling year, all belonging to the 1976–2012 period. Viral genetic diversity among HIV-infected children and adults with samples collected in Kinshasa between 2016 and 2018 was also calculated. We analyzed the genetic distance (number of base substitutions per site) or the average evolutionary divergence over all sequence pairs by using the Tamura-Nei model 93 (TN93)^77^, according to previous reports^67^. The rate variation among sites was modeled with a *gamma* distribution and analysis was conducted in MEGA6. We identified a recent transmission cluster in *pol* sequences showing maximum pairwise genetic distance lower than 0.01, according to previous reports^65–67^. TN93 was used because it is the most general nucleotide substitution model for which distances can be estimated directly from counts of nucleotide pairs in aligned sequences.

### Ethical aspects

The project was approved by the Human Subjects Review Committees at Monkole Hospital/University of Kinshasa (Kinshasa, DRC), University Hospital Ramón y Cajal (Madrid, Spain) and University of Navarra (Pamplona, Spain). Informed consent of all enrolled adults and of parents or guardians of enrolled children was obtained. All methods were carried out in accordance with relevant guidelines and regulations. Patients’ names were codified at sampling to maintain confidentiality.

### Statistical analysis

Differences in prevalence of HIV-1 variants were tested using T-test and *p values* < 0.05 were considered statistically significant. Descriptive statistical analysis was performed, median and interquartile range (IQR) was also calculated. Statistical analyses were conducted using Prism 6.0 software from GraphPad version 8.0.1 (San Diego, CA, USA).

## Supplementary information

Supplementary Information.
