# Supplementary material for: Current and historic HIV-1 molecular epidemiology in paediatric and adult population from Kinshasa in the Democratic Republic of Congo
Source: Sci Rep. 2020 Oct 28;10:18461. doi: 10.1038/s41598-020-74558-z (PMC7595211; doi:10.1038/s41598-020-74558-z)
Supplement: Supplementary file 1 — Supplementary Information. [file 41598_2020_74558_MOESM1_ESM.pdf]

CURRENT AND HISTORIC HIV-1 MOLECULAR EPIDEMIOLOGY IN PAEDIATRIC AND ADULT POPULATION FROM KINSHASA IN THE DEMOCRATIC REPUBLIC OF CONGO

Marina Rubio-Garrido[1]*, José María González-Alba[2]*, Gabriel Reina[3#], Adolphe Ndarabu[4], David Barquín[3], Silvia Carlos[5], Juan Carlos Galán[2] and África Holguín[1#].

[1] HIV-1 Molecular Epidemiology Laboratory, Microbiology and Parasitology Department, Hospital Ramón y Cajal-IRYCIS and CIBEREsp-RITIP, Madrid (28034), Spain.
[2] Virology Section, Microbiology and Parasitology Department, Hospital Ramón y Cajal-IRYCIS and CIBEREsp, Madrid (28034), Spain.
[3] Microbiology Department, Clínica Universidad de Navarra. Navarra Institute for Health Research (IdiSNA). Institute of Tropical Health, Universidad de Navarra (ISTUN), Pamplona (31008), Spain.
[4]Monkole Hospital, Kinshasa, the Democratic Republic of Congo.
[5]Department of Preventive Medicine and Public Health, Universidad de Navarra, Pamplona (31008), Spain. Navarra Institute for Health Research (IdiSNA). Institute of Tropical Health, Universidad de Navarra (ISTUN), Pamplona (31008), Spain.

* These authors contributed equally to this work. Author order was determined because this study belongs to the project of Rubio-Garrido M and González JM is a collaborator.

#Corresponding authors:

- Gabriel Reina
  E-mail: gabi@unav.es
  Tel. 948 255 400 ext. 5103
  Avda. Pio XII, 36. 31008 Pamplona (Spain)

- África Holguin
  Email: africa.holguin@salud.madrid.org
  Tel. 913 368 153
  Ctra. Colmenar Viejo Km 9,100. 28034 Madrid (Spain)

Supplementary Fig 1. Phylogenetic tree including 66 *pol* sequences ascribed to pure subtypes at *pol* from recently collected DBS from Kinshasa and 5,672 reference LANL sequences.

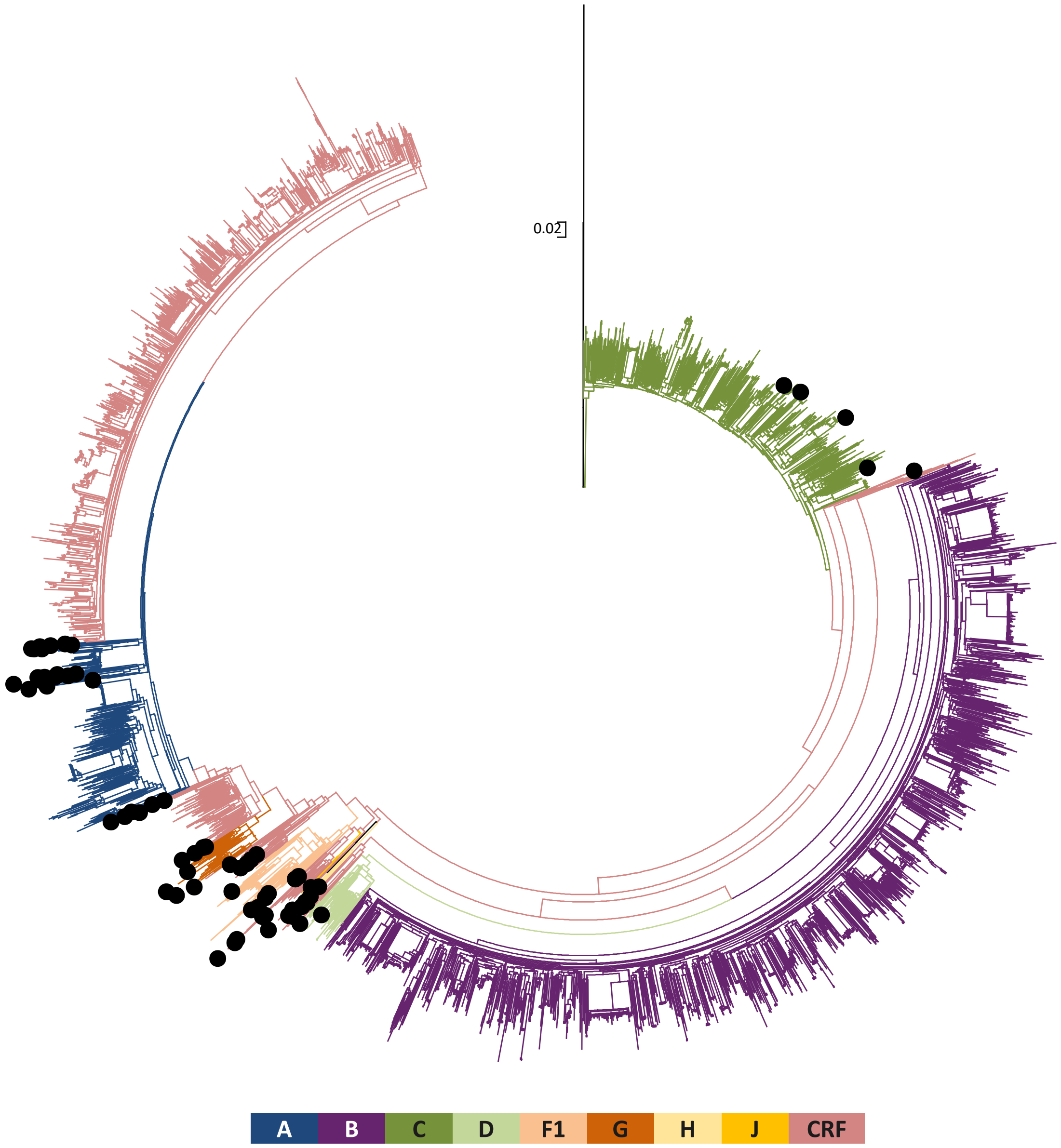

Supplementary Figure 1. Phylogenetic tree using partial *pol* sequences (1,127 nts) reconstructed by maximum-likelihood method with RAxML v8.0 using the general time reversible plus proportion of invariable sites plus gamma distribution parameter evolutionary model (GTR+I+G) generated with MEGA6 software (https://www.megasoftware.net/), as reported in method section. Only coloured subtypes are showed. Circles mark our 66 samples ascribed to pure HIV-1 subtypes at *pol* recently collected from Kinshasa (2016-2018). In blue, subtype A and sub-subtypes A1-A6.

Supplementary Figure 2. Transmission cluster number 1 identified by maximum-likelihood tree and GTR+I+G evolutionary model involving one child, one adolescent, and one woman in Kinshasa.

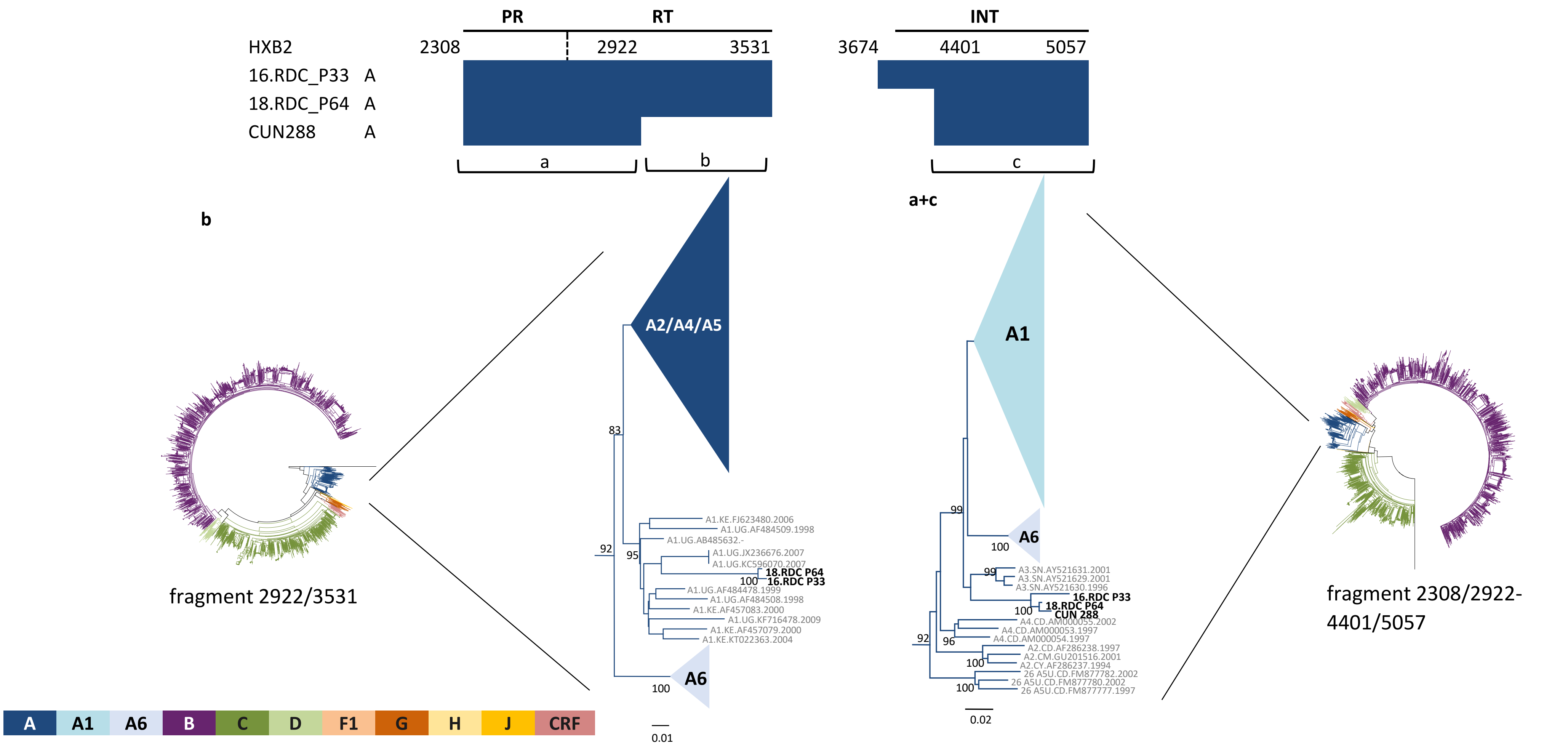

Legend Supplementary Figure 2. PhyML trees using partial *pol* sequence from 3 patients belonging to transmission cluster 1 including 4,090 *pol* reference sequences from DRC deposited in LANL assigned to HIV-1 pure subtypes. The figure was generated with MEGA6 software (https://www.megasoftware.net/). Epidemiological known data of subjects are reported in **Table 2**.

Supplementary Figure 3. Transmission cluster number 2 identified by maximum-likelihood tree and GTR+I+G evolutionary model involving two adults in Kinshasa.

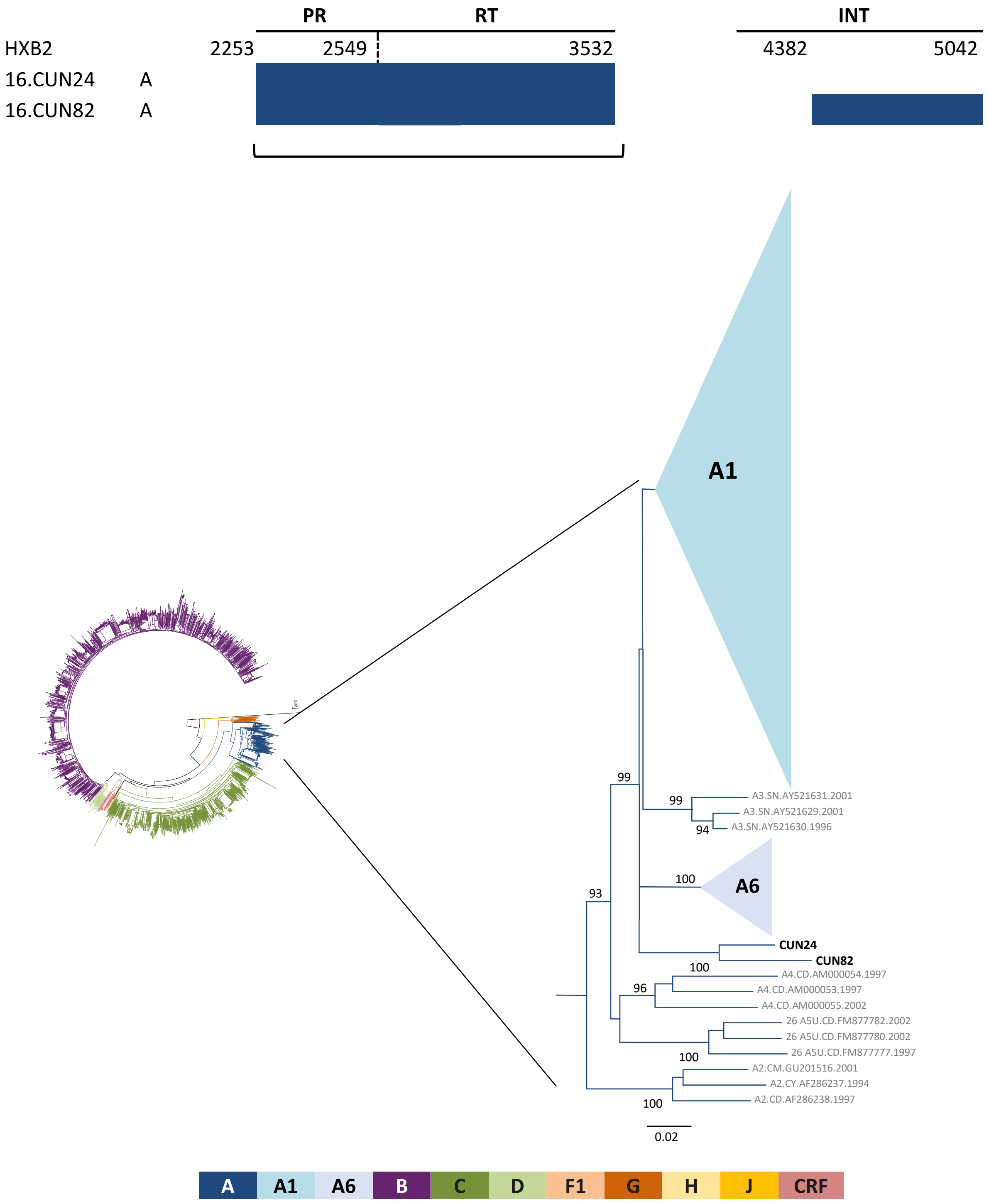

Legend Supplementary Figure 3. PhyML tree using partial *pol* sequence (1,279 nt) from patients CUN24 and CUN82 (transmission cluster 2) including 4,090 *pol* reference LANL sequences from DRC ascribed to HIV-1 pure subtypes**.** The figure was generated with MEGA6 software (https://www.megasoftware.net/). Epidemiological known data of subjects are reported in **Table 2.**

Supplementary Figure 4. Transmission cluster number 3 identified by maximum-likelihood tree and GTR+I+G evolutionary model involving two adults in Kinshasa.

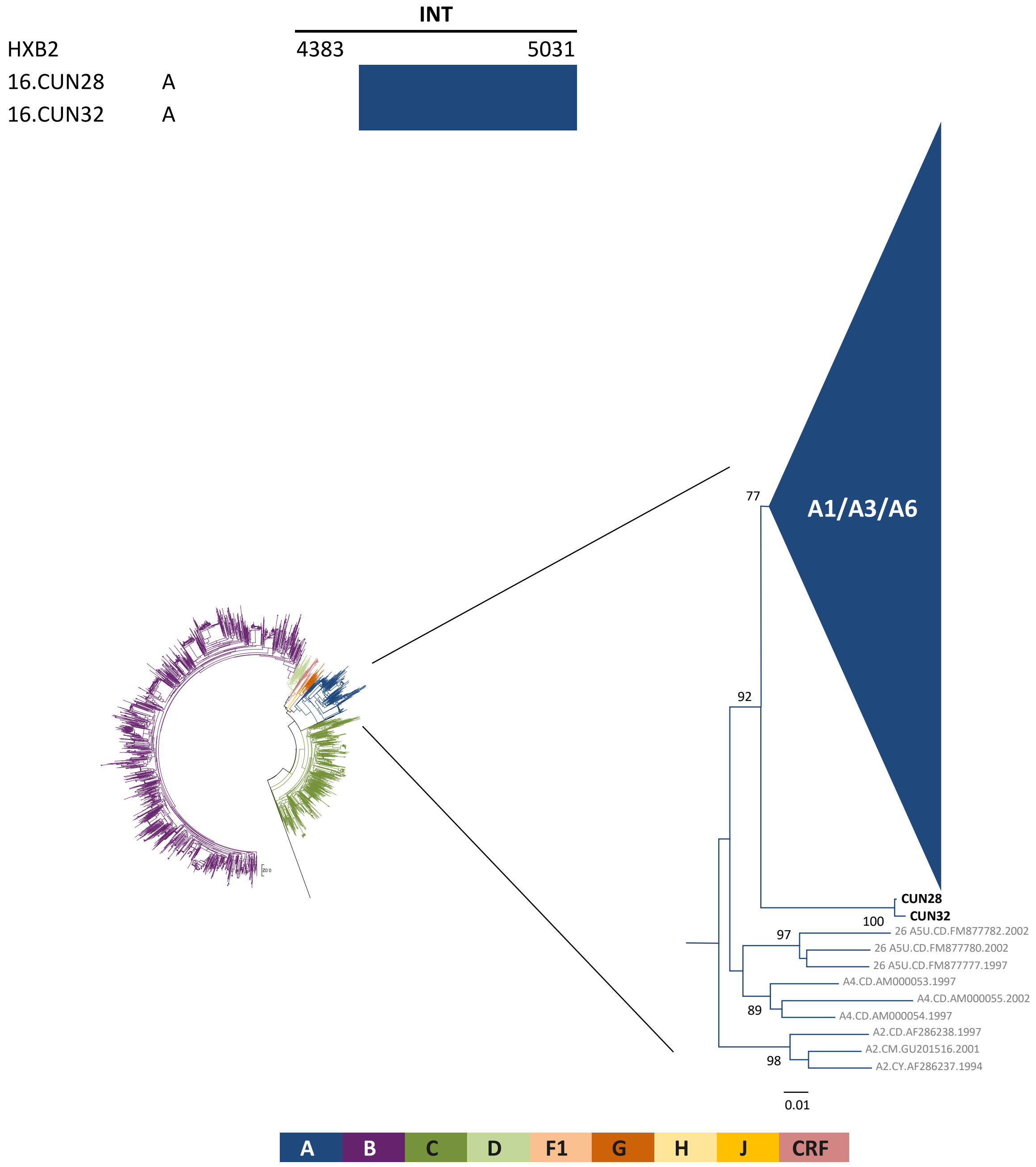

Legend Supplementary Figure 4. PhyML tree using partial *pol* sequence (648nt) from patients CUN28 and CUN32 (transmission cluster 3) including 4,090 *pol* reference LANL sequences from the DRC assigned to HIV-1 pure subtypes**.** The figure was generated with MEGA6 software (https://www.megasoftware.net/). Epidemiological known data of subjects are reported in **Table 2**.

Supplementary Figure 5. Transmission cluster number 4 identified by maximum-likelihood tree and GTR+I+G evolutionary model involving two children in Kinshasa carrying subtype H sequences at *pol*.

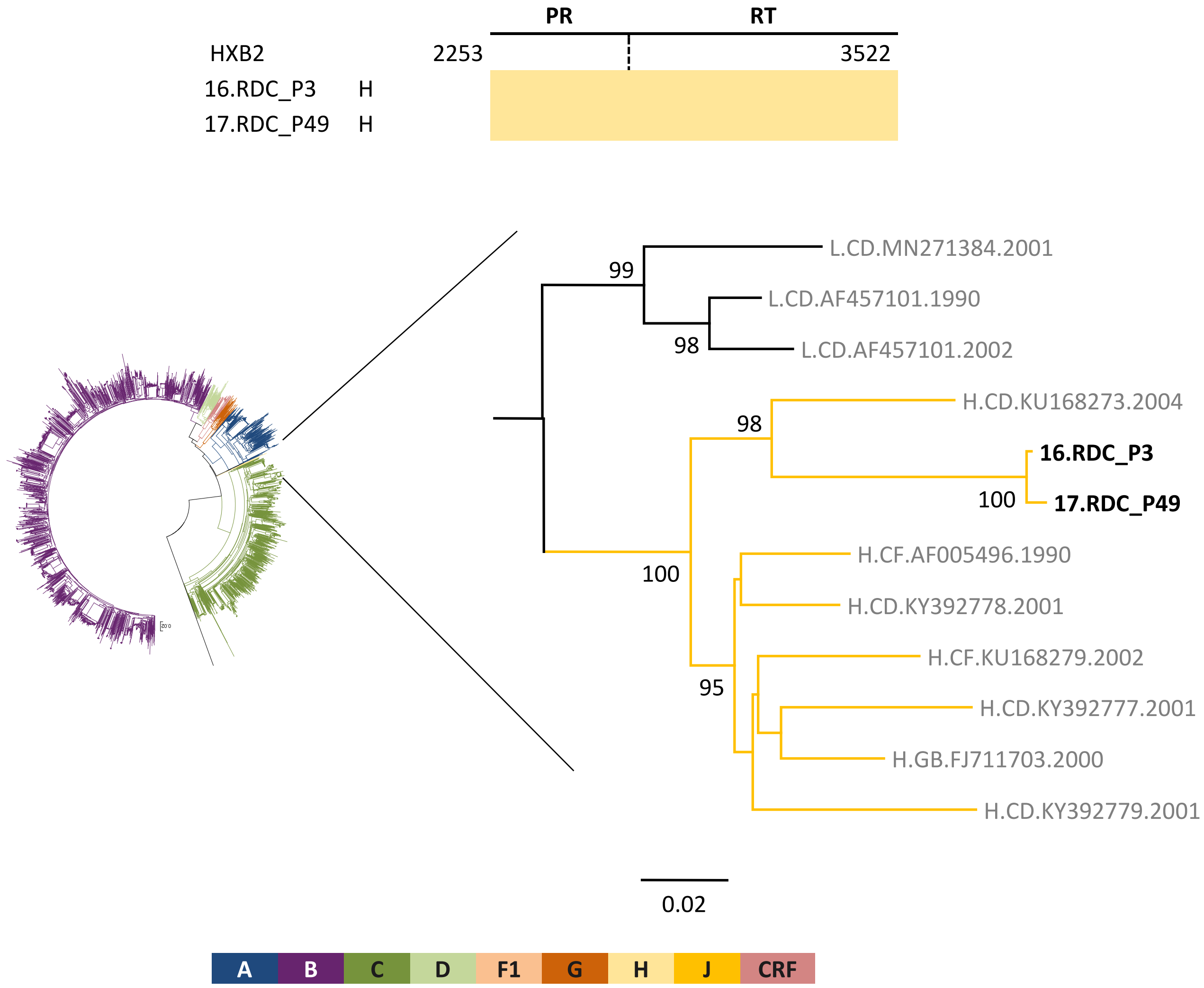

Legend Supplementary Figure 5. PhyML tree using partial *pol* sequence (1,269 nt) from 2 children included in transmission cluster 4 including 4,090 *pol* reference LANL sequences from the DRC assigned to HIV-1 pure subtypes**.** The figure was generated with MEGA6 software (https://www.megasoftware.net/). Epidemiological known data of subjects are reported in **Table 2.**

Supplementary Figure 6. Recombination transmission net from 17 involved paediatric patients from Kinshasa during 2016-2018.

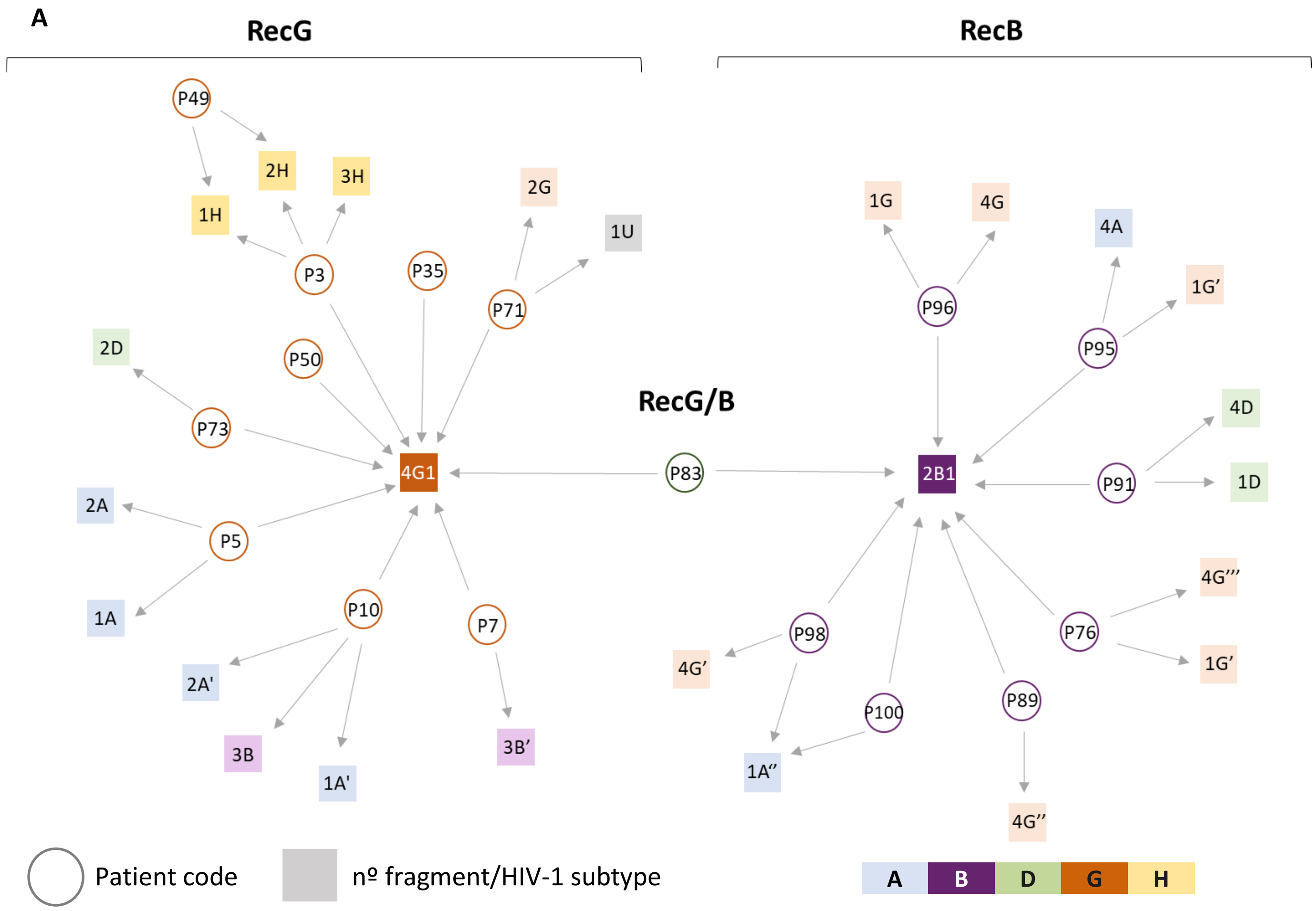

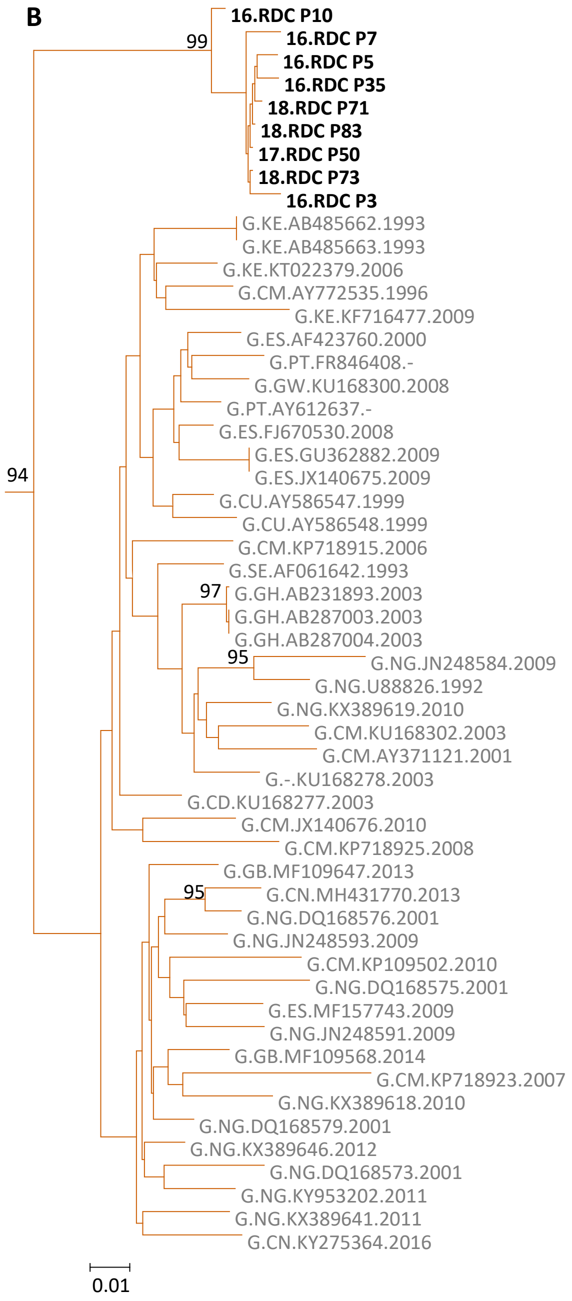

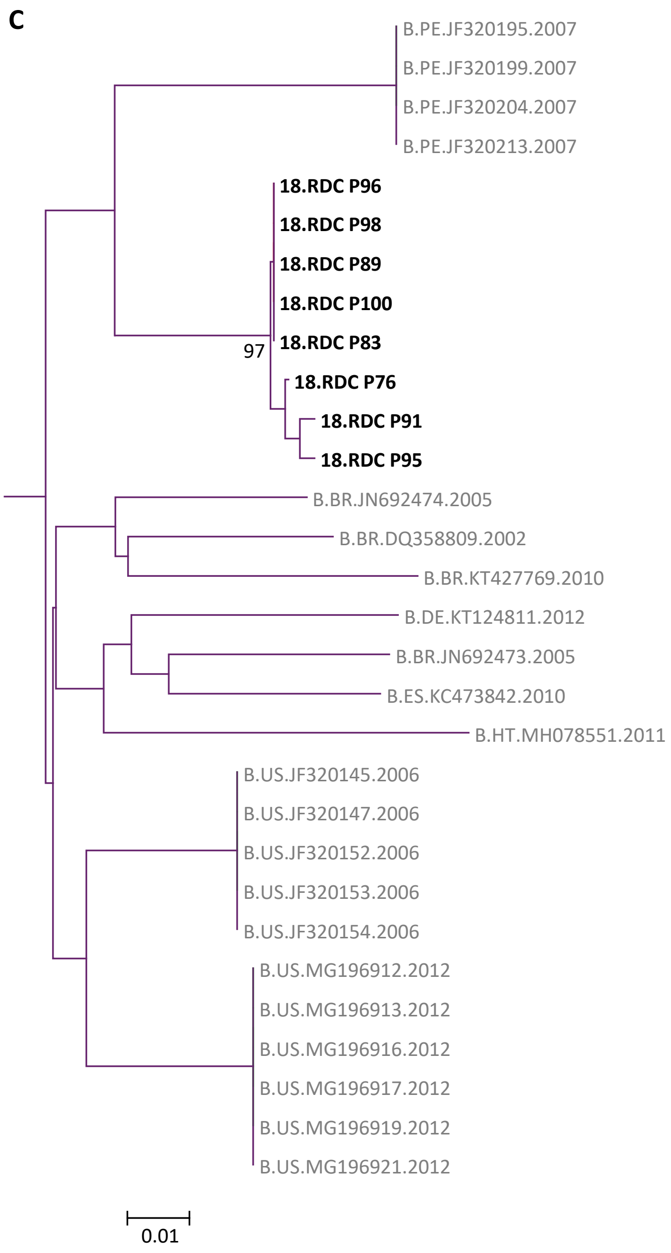

Legend Supplementary Figure 6. A. Recombination transmission net. **B.** PhyML tree of RecG, using partial *pol* sequence (615 nt, HXB2 4390-5005) from 9 children and adolescents including 4,090 *pol* reference LANL sequences from DRC ascribed to HIV-1 pure subtypes **C.** PhyML tree of RecB, using partial *pol* sequence (599 nt, HXB2 2935-3534) from 8 children and adolescents including 4,090 *pol* reference LANL sequences from DRC ascribed to HIV-1 pure subtypes. Coloured circles, patient´s *pol* sequences involved in recombination networks sharing *pol* subtype G (RecG) or/and B (RecB) fragments supported by bootstrap values >80% in PhyML trees. In coloured squares, numbers refer to analyzed *pol* fragment (numbering according to HXB2): 1, positions 2253-2934 (in protease); 2, positions 2935-3534 (in retrotranscriptase); 3, positions 3704-4389 (in retrotranscriptase); 4, positions 4390-5005 (in integrase). Letters refer to HIV-1 variant: A, subtype A; B, subtype B; D, subtype D; G, subtype G; H, subtype H; U, unknown. To distinguish the fragments of the same subtype that do not belong to the same ancestor, a super index is added. The figure was generated with MEGA6 software (https://www.megasoftware.net/). Epidemiological known data of subjects are reported in **Table 3**.

Supplementary Figure 7. Recombination event identified by maximum-likelihood (PhyML) tree and GTR+I+G evolutionary model involving viruses from 3 adult patients from Kinshasa.

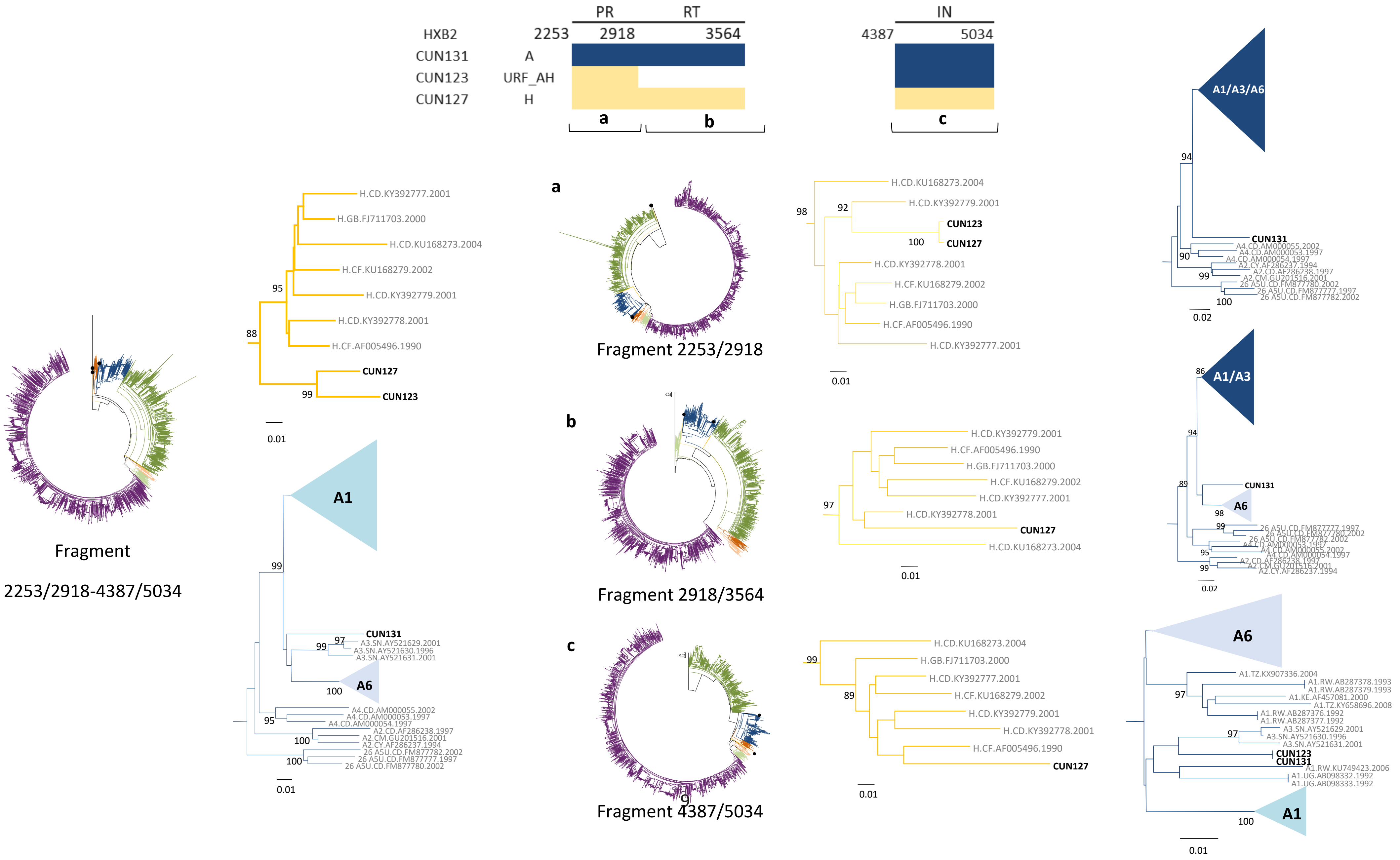

A	A1	A6	B	C	D	F1	G	H	J	CRF

Legend Supplementary Figure 7. PhyML tree using partial *pol* sequence (1,312 nt) from 3 adults including 4,090 *pol* reference LANL sequences from DRC ascribed to HIV-1 pure subtypes**.** The figure was generated with MEGA6 software (https://www.megasoftware.net/).

Supplementary Table 1. Summary of publications identifying HIV-1 variants in the DRC.

Study	DRC locations	Sampling year	Sample type	Population group	Subtyping method	No. seq	HIV genetic regions	Sequence submission to GenBank
**Potts 1993**[34]	Kinshasa	-	PBMC	Pregnant woman	Phy	14	*env (C2V3)*	No
**Mokili 1999**[35]	Kimpese	1988-1994	Blood, plasma and PBMC	Mothers and children	Phy	66	*gag (p17)*	No
**Vidal 2000A**[24]	Kinshasa, Bwamanda, Mbuyi-Maya	1997	PBMC	Sex worker, TB patients, pregnant women and blood donors	Phy	247	*env (V3-V5, gp160), gag (p24)*	Yes
**Vidal 2000B**[38]	Kinshasa	1997	PBMC	TB patient<br>Pregnant woman	Phy	2	*near-full-length genome*	Yes
**Triques 2000**[37]	-	-	-	-	Phy	1	*near-full-length genome*	Yes
**Yang 2001**[40]	Kinshasa	1985	plasma	Female sex workers	Phy	24	*gag (p24), env (C2V3, gp41)*	Yes
**Gao 2001**[41]	Kinshasa	1983	PBMC	AIDS patients	Phy	1	*gag, pol, env, nef*	Yes
**Mokili 2002**[36]	Kinshasa, Kimpese	1983<br>1990	PBMC	AIDS patient<br>12-month-old baby	Phy	2	*full genome*	Yes
**Kita 2004**[42]	Likasi	2001	PBMC	-	Phy	24	*pol (IN), env (C2V3,gp41)*	Yes
**Yang 2005**[39]	Kinshasa	1985<br>1999-2000	plasma	Female sex workers<br>STI and TB patients	Phy	24<br>83	*gag (p24)*<br>*env (C2V3, gp41)*	Yes
**Vidal 2005**[43]	Kinshasa, Mbuji-Mayi, Lubumbashi, Kisangani	2002	PBMC	-	Phy	288	*env (V3-V5)*	Yes
**Vidal 2006A**[44]	Kinshasa, Mbuji-Mayi, Lubumbashi, Kisangani	2002	PBMC	Sentinel population	Phy	70	*pol (PR,RT), env (V3-V5)*	Yes
**Vidal 2006B**[45]	Kinshasa	1997<br>2002	PBMC	TB patients	Phy	3	*near-full-length genome*	Yes
**Worobey 2008**[1]	Kinshasa	1960	lymph node biopsy	Adult female	Phy	1	*gag, pol, env*<br>*(small fragments)*	Yes
**Vidal 2008**[46]	Lubumbashi	2002	PBMC	-	Phy	1	*near-full-length genome*	Yes*
**Vidal 2009**[47]	Kinshasa, Mbuyi-Mayi, Lumbumbasi	1997<br>2002	PBMC	STI, ANC, AIDS, and TB patients	Phy	8	*near-full-length genome*	Yes

Study	Settings in DRC	Sampling year	Sample type	Population group	Subtyping method	No. seq	*HIV genetic regions*	Sequence submission to GenBank
**Niama 2009[48]**	-	1997	PBMC	-	Phy.	2	*full-length genome*	Yes*
**Huang 2009[49]**	-	1996	PBMC	Asymptomatic patients	Phy	2	*full-length genome*	Yes
**Djoko 2011[50]**	Kinshasa	2007	plasma	Military personnel	Phy	94	*pol (PR, RT)*	Yes
**Muwonga 2011[51]**	Kinshasa, Matadi, Lubumbashi, Mbuji-Mayi	2008	plasma	ART-treated patients<br>ART-naive patients	Phy	93	*pol (PR, RT)*	Yes
**Kamangu 2015[52]**	Kinshasa	2013-2014	plasma	ART-naive patients	Online subtyping tools	153	*pol (PR, RT)*	No
**Boillot 2016[53]**	Nord Kivu	2011-2012	DBS	ART-treated patients	Phy	55	*pol (PR, RT)*	Yes
**Rodgers 2017[29]**	Bandundu, Kananga	2001-2003	plasma	voluntary pregnant women	Online subtyping tools + Phy	172<br>14	*env (gp41)*<br>*full-length genome*	Yes
**Villabona 2017[54]**	Kinshasa, Mbuyi Mayi, Nord-Kivu	2008<br>2012	DBS or plasma	-	Phy	8	*full-length genome*	Yes
**Faria 2019[28]**	Kinshasa, Matadi, Mbuji-Mayi and Lubumbashi	2008	plasma	ART-treated patients	Phy	346	*pol (PR, RT)*	Yes
**Rubio-Garrido 2019[55]**	Kinshasa	2016	DBS	Children and adults	Phy	58	*pol (PR, RT, IN)*	Yes
**Yamaguchi 2020[10]**	Kananga	2001	plasma	-	Phy	1	*full-length genome*	Yes
**Kwon 2020[56]**	Kinshasa, Bukavu, Goma, Kananga, Kindu, Kikwit, Kisangani, Matadi, Lubumbashi, Mbandaka, Mbuji-Mayi	2012	DBS	Female sex workers	Online subtyping tools + Phy	60<br>145	*pol (PR, RT)*<br>*env (C2, V4)*	Yes
**Current study**	**Kinshasa**	**2016-2018**	**DBS**	**Children, adolescents and adults**	**Phy**	**165**	***pol (PR, RT, IN)***	**Yes**

Legend Supplementary Table 1. DRC, Democratic Republic of the Congo; DBS, dried blood spots; DPS, dried plasma spots; Dashes, unknown data; STI, sexually transmitted infection; ANC, antenatal clinic attendee; TB, tuberculosis; ART, antiretroviral therapy; AIDS; acquired immunodeficiency syndrome, PBMC, peripheral blood mononuclear cells; Phy, phylogenetic analysis of viral sequences; No, number; PR, protease; RT; retrotranscriptase; IN, integrase. GenBank, GenBank sequence database

(https://www.ncbi.nlm.nih.gov/genbank/); EMBL, EMBL Nucleotide Sequence Database (http://www.ebi.ac.uk/embl/). *Most papers submitted sequences to GenBank and 2 to EMBL database (with asterisks). The numbers in superindex indicate the reference of each article.
